# Myelosuppressive Conditioning Using Busulfan Enables Bone Marrow Cell Accumulation in the Spinal Cord of a Mouse Model of Amyotrophic Lateral Sclerosis

**DOI:** 10.1371/journal.pone.0060661

**Published:** 2013-04-08

**Authors:** Coral-Ann B. Lewis, John Manning, Christine Barr, Kyle Peake, R. Keith Humphries, Fabio Rossi, Charles Krieger

**Affiliations:** 1 Department of Biomedical Physiology and Kinesiology, Simon Fraser University, Burnaby, British Columbia, Canada; 2 The Biomedical Research Centre, University of British Columbia, Vancouver, British Columbia, Canada; 3 Terry Fox Laboratory/Department of Pathology, BC Cancer Agency, Vancouver, British Columbia, Canada; 4 Division of Neurology, Department of Medicine, Neuromuscular Disease Unit, VHHSC, Vancouver, British Columbia, Canada; University of Lyon, France

## Abstract

Myeloablative preconditioning using irradiation is the most commonly used technique to generate rodents having chimeric bone marrow, employed for the study of bone marrow-derived cell accumulation in the healthy and diseased central nervous system. However, irradiation has been shown to alter the blood-brain barrier, potentially creating confounding artefacts. To better study the potential of bone marrow-derived cells to function as treatment vehicles for neurodegenerative diseases alternative preconditioning regimens must be developed. We treated transgenic mice that over-express human mutant superoxide dismutase 1, a model of amyotrophic lateral sclerosis, with busulfan to determine whether this commonly used chemotherapeutic leads to stable chimerism and promotes the entry of bone marrow-derived cells into spinal cord. Intraperitoneal treatment with busulfan at 60 mg/kg or 80 mg/kg followed by intravenous injection of green fluorescent protein-expressing bone marrow resulted in sustained levels of chimerism (∼80%). Bone marrow-derived cells accumulated in the lumbar spinal cord of diseased mice at advanced stages of pathology at both doses, with limited numbers of bone marrow derived cells observed in the spinal cords of similarly treated, age-matched controls; the majority of bone marrow-derived cells in spinal cord immunolabelled for macrophage antigens. Comparatively, significantly greater numbers of bone marrow-derived cells were observed in lumbar spinal cord following irradiative myeloablation. These results demonstrate bone marrow-derived cell accumulation in diseased spinal cord is possible without irradiative preconditioning.

## Introduction

Clinical and experimental observations indicate that under certain conditions, bone marrow (BM)-derived cells (BMDCs) can transmigrate across the BBB and take up residence within the CNS. Studies employing BM chimeric rodents created using a myeloablative irradiation/BM transplantation paradigm have demonstrated that BMDCs migrate to and populate the CNS, and BMDC accumulation is significantly increased in affected areas of the CNS in murine models of neurodegenerative diseases including amyotrophic lateral sclerosis (ALS) [1], [2], [3], [4] and Alzheimer’s disease [5], [6], suggesting that BMDCs home to and/or expand at sites of neurodegeneration. In both the healthy and diseased murine CNS, the majority of BMDCs exhibit an immunophenotype consistent with CNS-associated macrophages, such as perivascular cells and other cell types, with a small proportion acquiring residence within the CNS parenchyma [4], [7].

A limitation in using BM chimeras to study cell migration into the CNS is that recipient mice are subjected to lethal levels of irradiation, which has been shown to induce changes in BBB permeability and incite an inflammatory response [8]. Furthermore, the intravenous injection of whole BM into the host circulation includes progenitor BMDC populations that under normal conditions would not be present in the bloodstream. Indeed, studies employing parabiosis, a technique that surgically joins the circulations of two genetically distinct mice resulting in peripheral blood cell (PBC) chimerism, have demonstrated that in the absence of irradiation and/or the nonphysiological presence of circulating BM progenitors, very few BMDCs are observed within the healthy or diseased CNS [9], [10].

Given the adverse side effects associated with lethal irradiation alternative conditioning regimens that enable BMDC accumulation in the CNS should be determined to improve the clinical potential of BMDCs as treatment modalities for neurological diseases. Busulfan (BU) is a clinically employed, well-established chemotherapeutic agent used to myelosuppress patients prior to receiving BM transplants. Myelosuppression using BU is an attractive alternative to irradiation particularly when transplanting autologous BM cells which would likely be the circumstances under which BMDCs would be used as treatment vehicles for neurological disease, as BU has only a minor effect on immune function, while irradiation leaves patients severely immunocompromised [11]. In mice, the myeloablative dose of BU has been reported to be between 135 to 150 mg/kg [12], [13] and some studies in which mice were treated with BU doses below this amount obtained variable levels of BM chimerism [11], [14]. Long-term BM chimerism using BU alone has been reported at 100 mg/kg [27].

Whether BU treatment enables BMDC accumulation in the CNS is currently a contentious issue, with two recent studies presenting conflicting results. Lampron and colleagues (2012) created BM chimeric mice using a combination of BU (80 mg/kg) and the immunosuppressant cyclophosphamide (CY; 200 mg/kg), followed by the intravenous injection with 2×10^∧^7 whole BM cells [28]. Although this treatment regimen resulted in high levels of peripheral blood cell (PBC) chimerism, no BMDC accumulation was observed in the naïve brain at 3 months post-BM transplant or in the hypoglossal nucleus following hypoglossal nerve transection [28]. Conversely, Capotondo and colleagues (2012) observed BMDC accumulation in the brains of BM chimeric naïve mice and a mouse model of lysosomal storage disease as early as 5 days following myelosuppression using 100 mg/kg BU and the transplantation of hematopoietic stem/progenitor (KLS) cells [27]. Given the disparity between these recent studies, further investigation into the efficacy of BU as a preconditioning agent that enables BMDC accumulation in the CNS should be undertaken.

To determine whether BU-induced preconditioning is capable of supporting sustained BM chimerism and permitting BMDC accumulation in the CNS, we created chimeric mice using donor BM harvested from mice that ubiquitously express green fluorescent protein (GFP). We performed BM transplants on control mice and a murine model of ALS that overexpresses mutant superoxide dismutase-1 (mSOD) to investigate whether the accumulation of donor-derived cells in the spinal cord was enhanced under neurodegenerative conditions. Significantly greater numbers of donor-derived cells were observed in lumbar spinal cord sections from mSOD mice at advanced stages of disease than in age-matched controls. Numbers of BMDCs in the spinal cord were significantly greater after myeloablation using irradiation than with BM chimerism following BU treatment at 60 or 80 mg/kg in mSOD and age-matched control mice when evaluated at advanced stages of disease (11–14 weeks post-transplant). Our study validates a myeloablative regimen using BU as an alternative to irradiation that permits stable BM chimerism and enables the accumulation of BMDCs in the diseased CNS. As BU-based myeloablation closely models some current clinical protocols, it should aid in the optimization of strategies for use of BMDC in CNS disease.

## Results

### Short-term Analysis of BM Reconstitution and BMDC Accumulation in the Healthy Spinal Cord Using BU

**Figure 1:**
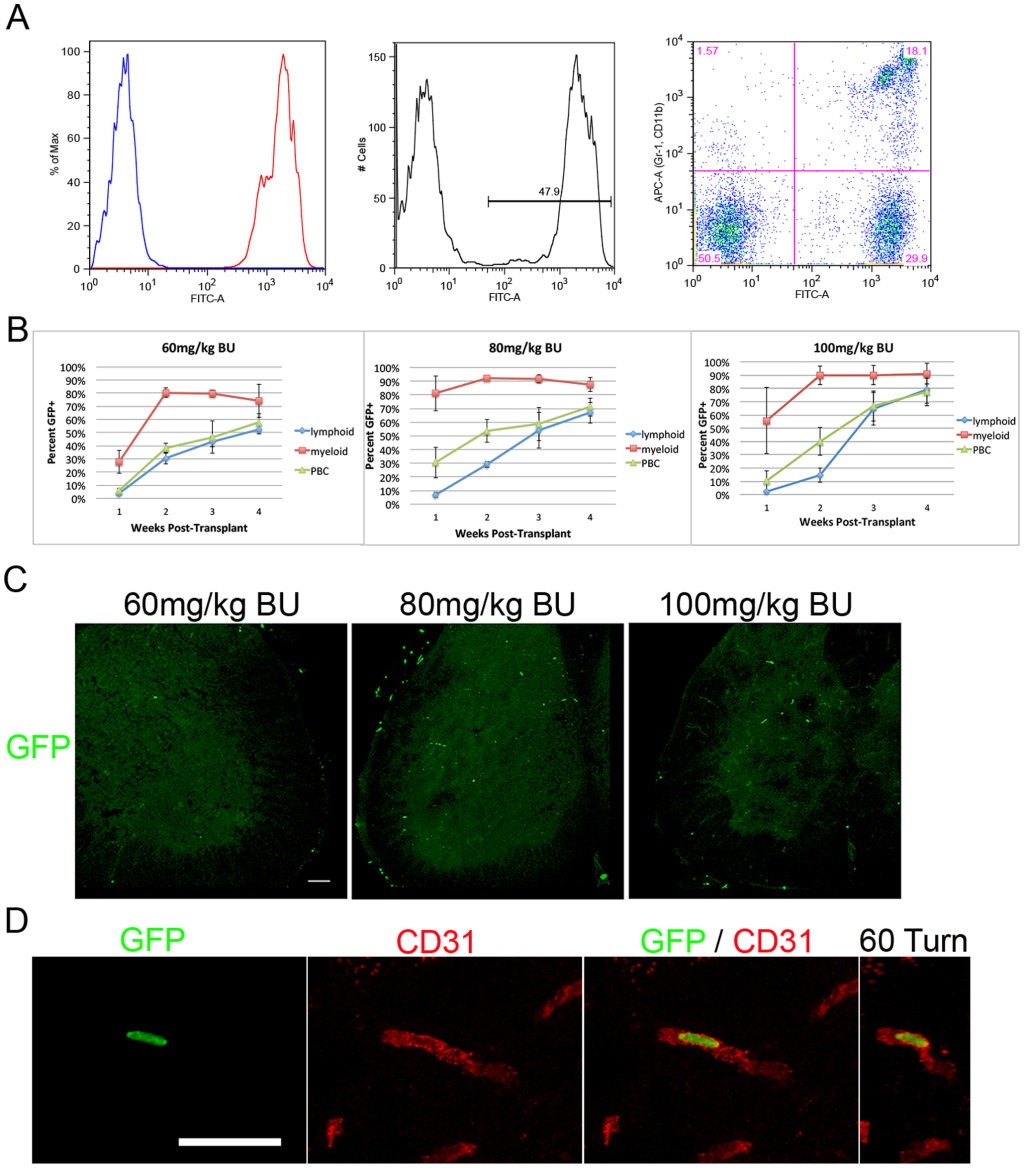
Short-term analysis of PBC reconstitution by donor cells using doses of 60, 80 or 100 mg/kg BU and GFP+ accumulation in control spinal cord. (**A**) Left: FACS plot of negative control (blue peak) and GFP+ control (red peak) blood. Centre: An example of a FACS plot of blood collected at 3 weeks post-BM transplant following treatment with 100 mg/kg BU. Roughly 48% of PBCs were GFP+. Right: Immunolabeling of blood for myeloid markers (CD11b-APC, Gr1-APC) indicate high level of donor chimerism in this blood cell population by 3 weeks post-transplant. (**B**) Levels of PBC and lymphoid chimerism increased over the 4-week observation period. Levels of chimerism in circulating myeloid cells increased at a rate higher than that of lymphoid cells. (**C**) By 4 weeks post-transplant, GFP+ cells were observed in the lumbar spinal cords of BM chimeric mice. Significantly greater numbers of GFP+ cells were observed in the spinal cords of mice treated with 80 or 100 mg/kg BU compared to mice treated with 60 mg/kg. Scale bar = 50 µm. (**D**) The majority of GFP+ cells in lumbar spinal cord exhibited a rod-shaped morphology and were associated with blood vessels immunolabelled with antibody to CD31. Scale bar = 50 µm.

To investigate the short-term level of PBC chimerism and BMDC engraftment within the healthy spinal cord following BU-induced myeloablation and BM transplantation, 8 week-old Bl.6 mice were treated with 60, 80 (n = 4 per group) or 100 mg/kg (n = 3) BU administered as fractionated doses of 20 mg/kg per day via intraperitoneal injection. Twenty-four hours after administering the final BU dose, mice were intravenously injected with 1.5×0^∧^7 GFP+ whole BM cells. Mice tolerated all three doses of BU well, exhibiting only minor reductions in weight during and following the treatment; all mice recovered well following BM transplantation. Beginning at 1 week post-transplant, weekly blood samples were collected and analysed via flow cytometry to determine levels of chimerism in circulating myeloid, lymphoid and total PBCs (figure 1A, B).

By 3 weeks post-transplant, total PBC chimerism was 46.5±2.5% (60 mg/kg BU), 58.7±12.2% (80 mg/kg BU), and 66.7±11.5% (100 mg/kg BU). Comparative levels of chimerism in circulating myeloid cells were 79.7±2.8%, 91.8±3.3%, and 89.6±7.2% for mice treated with 60, 80, and 100 mg/kg BU, respectively (figure 1B). This disparity between the total PBC and circulating myeloid cell chimerism can be attributed to the lack of immunosuppression elicited by BU, as the majority of circulating leukocytes are lymphoid cells, which are spared the cytotoxic effects of BU and have a relatively long half-life in the circulation [15]. Given the comparatively short half-life of circulating myelomonocytic cells, levels of chimerism in this population are more indicative of donor reconstitution of the BM compartment.

One month following BM transplantation, mice were sacrificed and spinal cords collected and analysed to assess levels of GFP+ BMDC accumulation. At all BU doses, limited numbers of GFP+ cells were observed in lumbar spinal cord sections which averaged 1.7±0.6 (mean±standard deviation; SD; n = 5 spinal cord sections per mouse), 10.0±2.4, and 7.3±1.7 for mice treated with 60, 80 and 100 mg/kg BU, respectively (figure 1C). In lumbar spinal cord sections from mice treated with 60 mg/kg or 80 mg/kg BU, all GFP+ cells were of a rod-shaped morphology and were found associated with CD31 positive spinal cord blood vessels when examined using confocal microscopy (figure 1D). These rod-shaped cells were similar in distribution and morphology to those observed by Audoy-Remus (2007), which were identified as a heterogenous population of myeloid cells that function to patrol blood vessels. The morphology of BMDCs in spinal cord sections from mice treated with 100 mg/kg BU was more heterogeneous, with a subset of cells exhibiting amoeboid and elongated morphologies possibly indicative of activation induced by toxic effects of BU.

Notably, GFP+ cells were infrequently observed in spinal cord sections from mice treated with 60 (n = 4), 80 (n = 4) or 100 mg/kg BU (n = 3) at 2 weeks post-transplant (data not shown). The results of this analysis indicate that limited numbers of BDMCs begin migrating to the vasculature in the naïve spinal cord between 2 and 4 weeks following BM transplantation. The extent of BMDC accumulation in spinal cord is positively correlated to the dose of BU administered, as the number of GFP+ cells in the lumbar spinal cord of mice treated with 80 or 100 mg/kg BU was significantly greater than in mice treated with 60 mg/kg BU (p<0.01).

### Stable, Long-term BM Reconstitution and BMDC Accumulation in the mSOD Spinal Cord Following Treatment with BU

**Figure 2:**
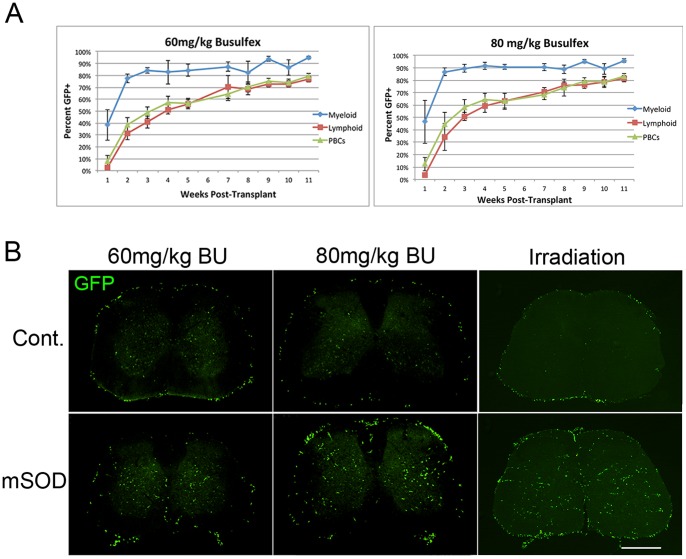
PBC reconstitution by donor cells and distribution of GFP+ cells in lumbar spinal cord. (**A**) Donor PBC reconstitution in mice treated with 60 mg/kg BU and 80 mg/kg BU was assessed weekly for 11 weeks post-transplant using flow cytometry. Levels of PBC chimerism achieved were similar between 60 mg/kg BU and 80 mg/kg BU treatment groups. Reconstitution of myelomonocytic cells was rapid in comparison to lymphoid populations, owing to the shorter half-life of circulating myelomonocytic cells and the minimal immunosuppressive effects of BU. Error bars represent standard deviation. (**B**) Distribution of GFP+ cells in control and mSOD lumbar spinal cord sections 11–14 weeks after transplantation following BU treatment or myeloablative irradiation. GFP+ cells accumulated in the grey and white matter of lumbar spinal cord sections, especially in the surrounding leptomeninges 11–14 weeks after transplantation following BU treatment or irradiation. Significantly greater numbers of BMDCs accumulated in mSOD lumbar spinal cords compared to age-matched controls using BU (60 or 80 mg/kg) or irradiative myelosuppression (p<0.05). Scale bar = 500 µm.

As no increase in donor cell engraftment was observed between mice treated with 80 and 100 mg/kg BU, we focussed on assessing PBC chimerism and BMDC accumulation in the in spinal cord over time using doses of 60 or 80 mg/kg BU. These doses were chosen based on our previous observations that very few BMDCs accumulated in the spinal cords of mice treated with 60 mg/kg BU, while significantly greater numbers accumulated in mice treated with 80 mg/kg BU at 1 month post-transplant. Age-matched control and mSOD mice were treated with either 60 mg/kg (n = 4 mSOD, 4 control) or 80 mg/kg BU (n = 4 mSOD, 4 control) and transplanted with 1.5×10^∧^7 GFP+ BM cells. Beginning at 1 week post-transplant and until mSOD mice reached advanced stages of disease blood was analyzed for the level of chimerism in circulating myeloid, lymphoid and total PBCs (figure 2A). Donor chimerism of myelomonocytic cells rose to asymptote at 3 weeks post-transplant, averaging 84.2±2.3% (mean ± SD) for mice treated with 60 mg/kg BU and 89.7±3.2% for mice treated with 80 mg/kg BU (figure 2A).

Lymphoid and total PBC chimerism continued to increase over the course of the 11-week observation period following BM transplantation, while levels of myeloid PBC chimerism remained constant (figure 2A). At 11 weeks post-transplant, total PBC chimerism averaged 81.1±3.4% for the 60 mg/kg BU treatment group and 83.3±2.6% for the 80 mg/kg BU treatment group, values that were not statistically different. Analysis of BM also revealed equivalent levels of donor chimerism for mice treated with 60 mg/kg BU (75.1±4.9%) and 80 mg/kg BU (74.5±4.9%). These levels of chimerism are similar to those previously reported in irradiated BM chimeric mice [2], [4] and demonstrate that sustained, high levels of BM chimerism can be achieved with BU in the absence of irradiation and other agents.

### Increased Numbers of BMDCs Accumulate in Diseased Spinal Cord

After BM transplantation, mSOD mice and age-matched controls were allowed to progress to advanced stages of disease at which point spinal cords were harvested. For both 60 mg/kg and 80 mg/kg BU treatment groups GFP+ cells were seen in both control and mSOD lumbar spinal cord sections. GFP+ cells were located throughout the grey and white matter, with large numbers of GFP+ cells observed in the leptomeninges surrounding the spinal cord (figure 2B). These observations are similar to those obtained using irradiated BM chimeric mice in which BMDC contribution to leptomeningeal macrophages is greater than to parenchymal macrophages/microglia within the spinal cord (figure 2B) [16], [17].

The number of GFP+ cells that accumulated in control mice averaged 7.7±3.8 (range 3.8±1.7 to 11.9±2.8) cells per lumbar spinal cord section in 60 mg/kg BU treated control mice, and 12.8±4.5 (range 8.8±1.0 to 19.0±1.7) in mice treated with 80 mg/kg BU. In mSOD mice treated with 60 mg/kg BU, the mean number of GFP+ cells per lumbar spinal cord section was 46.2±18.6 (range 31.7±1.8 and 73.3±3.0). In the 80 mg/kg BU mSOD mouse treatment group, the mean number of GFP+ cells was 74.3±44.5 (range 25.2±1.6 to 130.9±10.6) per lumbar spinal cord section. Thus, significantly greater numbers of GFP+ BMDCs accumulated in the spinal cords of mSOD mice compared to age-matched controls at both the 60 mg/kg and 80 mg/kg BU dose (p<0.05). However, while there was a clear trend for mSOD and control mice treated with 80 mg/kg BU to have increased numbers of GFP+ cells per lumbar spinal cord section compared to those treated with 60 mg/kg BU, significant variability within the groups prevented this difference from reaching statistical significance.

To compare the accumulation of BMDCs in the spinal cord of BM chimeric mice created using BU and irradiative myeloablation, control (n = 3) and age-matched mSOD (n = 3) mice were subjected to 10 Gy of radiation and transplanted with 5×10^∧^6 GFP+ BM cells. Irradiated BM chimeric mice exhibited ∼80% PBC chimerism at 3 weeks-post transplant, a level of PBC chimerism comparable to that of mice myelosuppressed using BU. However, 80% PBC chimerism was obtained in irradiated chimeras more rapidly (i.e. 3 weeks post-transplant) than in BU myelosuppressed mice, given the cytotoxic effects of irradiation on lymphocytes. Spinal cords were collected and analysed for GFP+ cell accumulation in lumbar spinal cord sections once mSOD mice reached advanced stages of disease. Consistent with previous results, significantly greater numbers of GFP+ cells were observed in the mSOD lumbar spinal cord sections (422.6±159.1 GFP+ cells per section) than in control spinal cord sections (63.3±17.2 GFP+ cells per section; p<0.05) [2], [4]. Comparatively, significantly greater numbers of BDMCs were observed in both the irradiated control and mSOD lumbar spinal cord compared to the BU treated groups at both doses studied (p<0.01).

Our results demonstrate that while only minimal numbers of BMDCs are present in spinal cords of control mice treated with BU, BMDC accumulation is significantly enhanced in the diseased spinal cord, indicating there is increased migration and/or BMDC expansion at sites of neurodegeneration. However, significantly greater numbers of BDMCs accumulate in the mSOD and control spinal cord under the conditions of irradiation/BM transplantation compared to BU-induced myelosuppression/BM transplantation at the BU doses used in this study. As it was observed that there was a trend towards increasing numbers of BMDCs engrafted in spinal cord following treatment with 80 mg/kg compared to 60 mg/kg BU, it is possible that increasing the BU dose would increase the levels of BMDCs observed in the spinal cord. However, it may also increase the cytotoxicity of the preconditioning treatment.

### Analysis of BMDC Morphology and Immunophenotype

**Figure 3:**
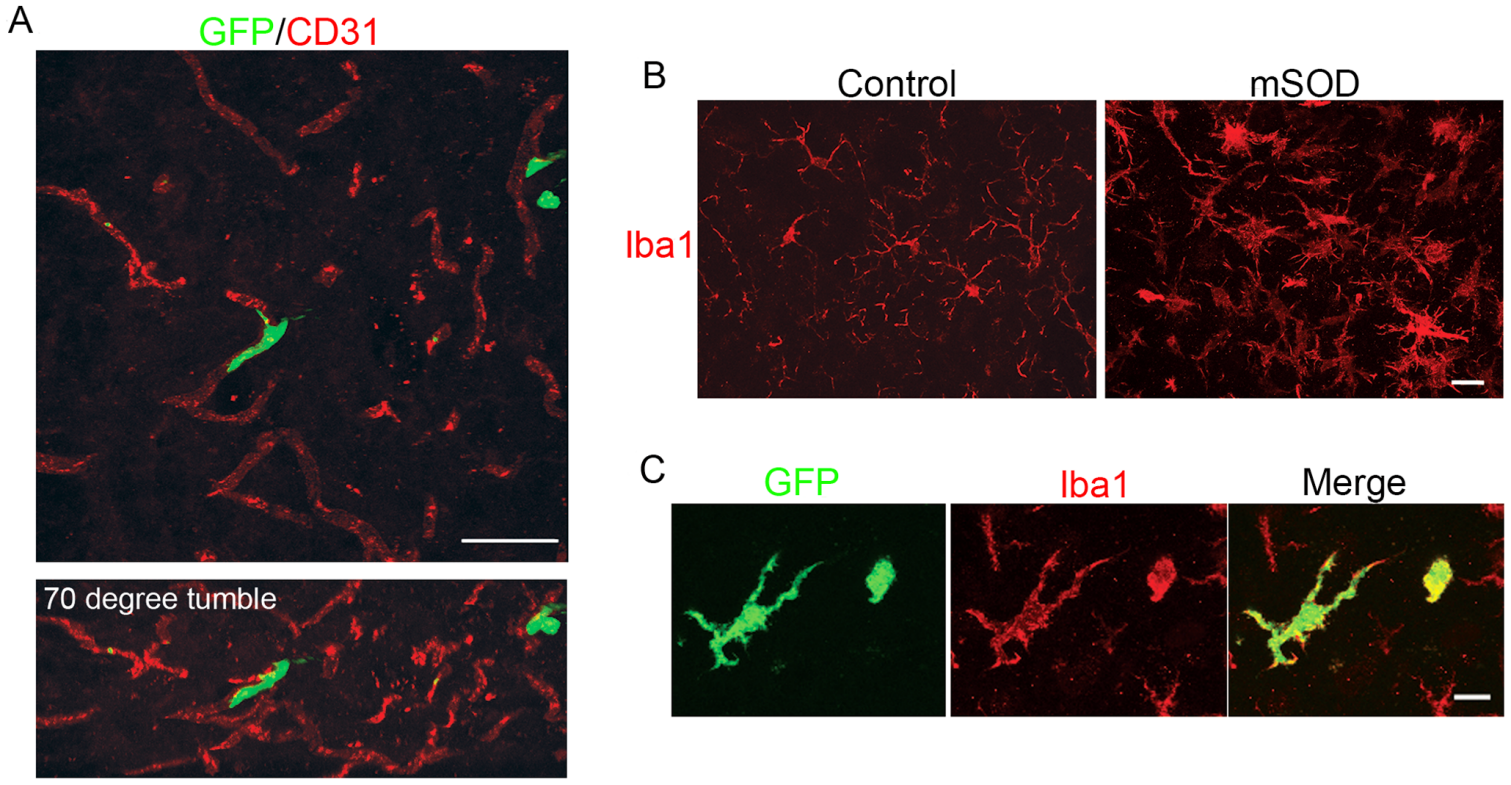
BMDCs in the spinal cord acquire the anatomical location and immunophenotype of various CNS-associated macrophages. (**A**) Upper Panel: GFP+ cells with an elongated morphology were located near blood vessels within mSOD and control lumbar spinal cord sections. Lower Panel: 3D-rendered confocal image rotated backwards at 70 degrees indicating the elongated GFP+ cell is located on the abluminal side of the blood vessel. Scale bar = 50 µm. (**B**) Control and mSOD lumbar spinal cord sections immunolabelled with antibody to Iba1. Increased numbers of microglia were observed in the mSOD spinal cord and exhibited an active morphology characterized by hypertrophied cell bodies and retracted, thickened cellular processes. Scale bar = 10 µm (**C**) GFP+ cell possessing stellate morphology immunolabelled with antibody to Iba1 identifying these cells as BM-derived parenchymal macrophages. Scale bar = 10 µm.

In an attempt to characterize BMDCs within the spinal cord of BU treated mice, BMDC morphology was classified as being round, rod, amoeboid, stellate or elongated in shape, as described in previous work (table 1) [7], [4]. Differences in the frequency with which defined cell morphologies of BMDCs are observed likely reflect the differing state of the spinal cord microenvironment induced by disease. Elongated BMDCs were often found in a perivascular location and exhibited low levels of Iba1 expression, indicating these BMDCs acquired the phenotype of perivascular macrophages (figure 3A) [7]. In mSOD mice, advanced disease stages are associated with widespread microgliosis within the spinal cord and increased expression of inflammatory mediators that likely influence the differentiation of BMDCs that accumulate within the spinal cord [18]. Indeed, we observed a greater proportion of BMDCs in mSOD mice acquiring a stellate morphology compared to control mice, although the absolute numbers of these cells are low. Similarly, differences in the cytokine milieu within the spinal cord evoked by irradiation and BU treatment could also affect the differentiation of BMDCs that accumulate in the spinal cord. Specifically, cells with an amoeboid morphology constituted the greatest proportion of BMDCs observed in both control and mSOD irradiated BM chimeric mice. This observation is in agreement with previous reports indicating irradiation-induced changes in the cytokine milieu within brain parenchyma culminates in alterations in the morphology of microglia [29].

**Table 1:** Morphological proportions of BMDCs in control and mSOD lumbar spinal cord^1^.

	Averagecells/section	round	rod	amoeboid	stellate	elongated
		GFP cells	GFP cells	GFP cells	GFP cells	GFP cells
60 mg/kg BU control (n = 4)	7.7±3.9	10.1±6.3%	65.5±8.3%	1.8±0.4%	2.0±3.2%	20.6±14.2%
						
						
60 mg/kg BU mSOD (n = 4)	46.2±18.6	10.9±8.0%	29.4±2.8%	2.5±0.8%	18.0±9.0%	39.3±5.5%
						
						
80 mg/kg BU control (n = 4)	12.8±4.5	4.9±3.4%	53.1±4.4%	1.7±0.5%	3.6±5.0%	36.8±10.8%
80 mg/kg BU mSOD (n = 4)	74.3±44.5	4.8±2.3%	29.8±4.3%	2.0±1.6%	22.0±10.3%	41.5±6.3%
						
						
Irradiation control (n = 3)	63.3±17.2	23.4±1.7	17.3±1.1%	42.8±7.0%	0.2±0.0%	16.3±1.2%
Irradiation mSOD (n = 3)	422.6±159.1	16.8±4.5	15.2±4.2%	44.2±32.3%	3.4±0.2%	20.3±8.0%

1 GFP+ BMDCs in lumbar spinal cord sections collected from mSOD and age-matched control mice at advanced stages of disease quantified and classified by morphology; values are reported as mean ± standard deviation.

Immunolabelling of lumbar spinal cords sections with antibody to the mature microglia/macrophage marker Iba1 revealed widespread microgliosis in the lumbar spinal cord of mSOD mice, as indicated by increased numbers of Iba1+ cells and Iba1 labelling intensity compared to control mice (figure 3B). Microglia in mSOD spinal cords exhibited an activated morphology characterized by hypertrophied cell bodies and retracted, thickened processes, while in control spinal cords their morphology was consistent with that described for resting microglia, as indicated by small cell bodies and thin, highly ramified processes (figure 3B) [19]. The proportion of GFP+ donor cells that expressed Iba1 was quantified as a function of cell morphology in control and mSOD mice; these values were then averaged over mSOD and control groups within the 60 mg/kg and 80 mg/kg BU treatment groups. Averaged across all morphological classes, the proportion of BMDCs that expressed Iba1 was 58.4±23.5% and 75.7±23.8% (mean ± s.d.) in control mice treated with 60 and 80 mg/kg BU, respectively. These numbers likely under represent Iba1+ BMDCs as immunohistochemical analyses are often limited by a lack of sensitivity in labelling the low-level antigen expression that would be expected in healthy spinal cord. Indeed, in mSOD mice the vast majority of GFP+ cells were clearly positive for Iba1 treated with BU (80.5±4.8% and 90.3±4.9%, in mice treated with 60 mg/kg and 80 mg/kg BU, respectively). These results indicate that the large majority of BMDCs acquire a macrophage/microglial phenotype within the spinal cord.

Iba1 expression varied with GFP+ cell morphology, with 100% of elongated, stellate and amoeboid cells in mSOD spinal cord from both BU treatment groups exhibiting Iba1-positive immunolabelling. The stellate-morphology and Iba1+ immunolabelling of GFP+ cells together identify these cells as BM-derived spinal cord parenchymal macrophages (figure 3C). Rod-shaped cells were found in association with blood vessels and exhibited variable immunolabelling with antibodies to Iba1, CD11b and Gr-1. Some of these Iba1-negative GFP+ cells were similar immunophenotypically and morphologically to cells described by Audoy-Remus and colleagues [20], which were identified as monocytes or granulocytes that reside within the lumen of blood vessels and function to patrol the CNS vasculature. Many round-shaped GFP+ cells in control and mSOD mice morphologically resembled T-cells, with a thin layer of cytoplasm surrounding a large nucleus; this was confirmed by immunolabelling for the pan-T-cell antigen CD3 which positively labelled the vast majority of round GFP+ cells.

As BU is known to have neurotoxic effects in patients and animals at high-doses [30] and the presence of CD8+ T-cells within the CNS is associated with neuronal injury or neurological disease [22], we sought to determine whether the doses of BU used in this study altered T-cell number in naïve and mSOD spinal cord. Control mice treated with 60 mg/kg BU and 80 mg/kg BU exhibited averages of 4.9±3.8 and 3.7±1.9 T-cells per lumbar section, respectively. Both values were not significantly different from the average number of T-cells in similarly aged, untreated control mice (n = 3), which averaged 4.7±1.2 cells per lumbar spinal cord section. The average number of T-cells per mSOD lumbar spinal cord section was 15.8±5.9 cells/section for mice treated with 60 mg/kg BU and 17.1±7.8 cells/section for mice treated with 80 mg/kg BU. These values were not significantly different from those observed in untreated advanced mSOD mice (n = 3), which were found to have an average 24.4±6.1 T-cells per lumbar spinal cord section.

T-cells within lumbar spinal cord sections were further classified by double-immunolabelling with antibodies to CD3 and CD8, a marker for cytotoxic T-cells. In mSOD treated with 60 mg/kg or 80 mg/kg BU, ∼30% and 40% of T-cells were CD8+, identifying these cells as cytotoxic T-cells. These values are in line with previous reports, which demonstrated that at advanced stages of disease, cytotoxic T-cells accumulate in the spinal cord of mSOD mice [21], [3]. The majority (>80%) of T-cells within lumbar spinal cord sections of control mice treated with 60 or 80 mg/kg BU were CD8 negative. This observation is in agreement with previous reports indicating that CD8+ T-cells are rarely observed in the healthy CNS [22]. BU readily crosses the BBB and in humans, side effects include seizures and altered mental development in children [23]. The absence of CD8+ T-cells within the spinal cord of control mice treated with BU suggests that treatment with BU at the doses employed in this study does not induce significant toxicity or neuroinflammatory responses.

These results demonstrate that BMDCs accumulating in the spinal cord of control and mSOD mice following Bu-induced myeloablation and BM transplantation primarily differentiate into CNS-associated (i.e. perivascular, meningeal) macrophages, with a small proportion taking up residence within the parenchyma proper. Furthermore, at doses of 60 and 80 mg/kg, BU does not evoke a substantial neuroinflammatory response in naïve animals, as indicated by the resting morphology of microglia and a T-cell repertoire comparable to that of untreated mice.

## Discussion

Previous studies employing BM chimeric mice created by lethal irradiation and BM transplantation have demonstrated that under these conditions, BMDCs accumulate in the CNS [16], [7]. In BM chimeric models of neurodegenerative disease including the mSOD mouse model of ALS, significantly greater numbers of BMDCs accumulate in the diseased CNS compared to that of healthy controls, suggesting BMDCs migrate to or expand at sites of neurodegeneration and have the potential to function as treatment vehicles for neurodegenerative disorders [2], [4]. We have demonstrated that levels of BM chimerism similar to those obtained with irradiation can be achieved using the clinically established chemotherapeutic agent BU. Furthermore, while BMDCs accumulate in the spinal cord of mSOD mice treated with BU, only very limited numbers were observed in the spinal cords of similarly treated control mice, demonstrating that this treatment permits only minimal BMDC accumulation in the spinal cord under steady-state conditions.

Overall, our results are in agreement with those recently reported by Capatondo and colleagues (2012) who also found that myeloablation using BU alone prior to BM transplantation led to entry and/or expansion of donor-derived BMDCs in the CNS of the recipient and support the notion that BU effectively conditions the CNS for BMDC engraftment [27]. Our work confirms the potential utility of BU as a myeloablative agent. While the accumulation of BMDCs in the CNS was greater following irradiative versus BU-induced myeloablation, the fact that the BU treatment was better tolerated than irradiation may have clinical application.

### Creation of BM Chimeras

Although the myeloablative dose of BU has been reported to be 135–150 mg/kg [12], [13], we found that BU at doses of 60 or 80 mg/kg produced levels of PBC chimerism similar to those previously obtained using irradiative myeloablation (∼80%) [2], [4]. However, the time required for achieving high levels of PBC chimerism in BU-treated mice was longer than that observed in irradiated mice. The reason for this delay may be that compared to irradiation, BU is only mildly immunosuppressive and T-lymphocyte populations in the treated recipient are largely spared [11]. Given that lymphoid cells constitute a large proportion of the total number of circulating leukocytes (>80%) [15] and have a long half-life in the circulation, overall PBC chimerism in BU treated mice at 3 weeks post-transplant was less than that previously observed in irradiated chimeras. However, in agreement with previous studies, levels of chimerism in circulating myelomonocytic cells, which due to their relatively short half-life more immediately reflect hematopoietic stem cell activity in BM, demonstrated rapid reconstitution with donor cells within the first 3 weeks of BM transplantation [13]. Eventually, total donor-derived cells in BU treated recipients increased to levels similar to those seen in irradiated BM chimeras (∼80% of PBCs) and persisted over the 11 weeks following BM transplant until mSOD mice reached advanced stages of disease.

### BMDCs Accumulate in the Spinal Cords of Control and mSOD BM Chimeric Mice

All animals which developed peripheral blood chimerism in our experiments also exhibited BMDCs within the spinal cord. We did not specifically assess BMDC entry into the brainstem or brain, but qualitatively our data are similar to the recently reported results of Capotondo et al. (2012) showing BMDC entry into brain following BU myeloablation and BM transplantation [27]. A direct comparison between the numbers of BMDCs found in the spinal cords of BU treated animals here, and those observed by Capotondo et al. cannot be made, as levels BMDCs that accumulated in the brain in their study were assessed using flow cytometry, which does not allow to exclude with certainty cells present in the meninges, and control and diseased brains were pooled [27]. A recent study by Lampron and colleagues employing myeloablation induced by BU with CY prior to BM transplantation reported that BMDCs did not accumulate in the brainstem, either with or without hypoglossal nerve axotomy [28]. The reason BMDC cells were not observed in the CNS in that study is unclear [28].

While only limited numbers of BMDCs were observed in the spinal cords of healthy mice treated with 60 mg/kg or 80 mg/kg BU, significantly greater numbers of BDMCs accumulated in the spinal cord of mSOD mice in both treatment groups. At disease end-stage, GFP+ cell numbers were found to be 5 to-10 fold higher than in age-matched controls, consistent with previous results [2]. Similar results were obtained in irradiated/transplanted BM chimeric mSOD mice, although the absolute numbers of BDMCs that accumulated in the spinal cords of mice treated with BU was lower than that observed in irradiated mSOD mice. This observation, together with the observation that long term peripheral blood chimersm was equivalent with both protocols, suggests that while the myeloablation provided by BU in bone marrow is comparable with that elicited by irradiation, its effects on the CNS are milder but still sufficient to allow BMDC accumulation in the diseased spinal cord.

### BMDC Morphology and Immunophenotype

Categorization of GFP+ cells by their morphology provides insight into the identity and function of these cells. The stellate morphology is characteristic of parenchymal macrophages/microglia, while elongated morphology is associated with perivascular macrophages that reside between the basal lamina of blood vessels and the glia limitans [7], [25]. An amoeboid morphology is seen with activated, phagocytic microglia [19], as well as with perivascular macrophages [7]. Rod-shaped cells have been described as resident monocytes or granulocytes that reside within the lumen of blood vessels where they function to patrol the vasculature [20]. The majority of round GFP+ cells were identified as T-cells by immunolabelling with antibody to CD3.

The number of T-cells observed in the lumbar spinal cords of control and mSOD mice in both the 60 and 80 mg/kg BU treatment groups were not significantly different from those observed in untreated mice. In agreement with previous reports, immunolabeling with CD3 and CD8 antibodies indicated that cytotoxic T-cells accumulate in the spinal cords of mSOD mice at advanced stages of disease [21], [3]. Only rare CD8+ T-cells were observed control lumbar spinal cord sections; this was expected as these cells are infrequently observed in the healthy CNS [22].

A greater proportion of GFP+ cells in mSOD spinal cord expressed Iba1 compared to control spinal cord in both the 60 mg/kg BU and 80 mg/kg BU treatment groups. This disparity between groups may be due to differentiation of BMDCs into macrophages or the expansion of donor-derived myelomonocytic cells in response to disease-induced environmental stimuli in the mSOD spinal cord. Notably, in control and mSOD spinal cords, all GFP+ cells having a stellate morphology also expressed Iba1 indicating that these cells differentiated into parenchymal microglia/macrophages.

These results indicate that treatment with BU enables the contribution of BMDCs to the parenchymal macrophage populations within the spinal cord under neurodegenerative conditions.

### Conclusion

The results of this study show that preconditioning with BU permits the engraftment of BMDCs in both the naïve and diseased spinal cord. Given that BDMC accumulation is significantly increased in the spinal cords of mSOD mice compared to controls, BU-mediated myeloablation could be useful to probe the clinical potential of BMDCs as treatment vehicles in neurological disease.

## Methods

### Ethics Statement

Mice used in this study were provided food and water *ad libitum* and all efforts were made to minimize suffering. All protocols related to the use of animals in this study were reviewed and approved by the University Animal Care Committee of Simon Fraser University (UACC; permit numbers 1036K-12 and 923K-09) and were in compliance with the Canadian Council on Animal Care, the NIH Guide for the Care and Use of Laboratory Animals, and the EEC Council Directive.

### Animals

Transgenic C57/B6 mice that over-express the human SOD1 (G93A) missense mutation (mSOD) were bred from progenitor stock obtained from Jackson Laboratories (Bar Harbour, ME). Mice were maintained as heterozygotes by breeding mSOD males with non-transgenic females and progeny were genotyped for mSOD transgene using a protocol established by Gurney and colleagues [26]. The mSOD mice develop progressive motoneuron degeneration, culminating in muscle atrophy and eventually hind limb paralysis [26]. Age and sex-matched, non-transgenic mice were used as controls and sacrificed at the same time point as mSOD animals.

Mice that ubiquitously express green fluorescent protein (GFP) under the control of the B-actin promoter (C57BL/6; GFP/CD45.2) were obtained from Dr. I. Weissmann and were bred and maintained as heterozygotes at the Animal Research Facility (ARC) at Simon Fraser University (SFU). GFP-expressing mice aged 8 weeks to 6 months served as BM donors and BM was harvested by flushing femurs and tibiae with sterile PBS using a syringe; BM cells were quantified using a haemocytometer. Male BM recipients received only male BM while female recipients received only female BM to avoid any graft-versus-host effects.

### Irradiative Myeloablation

Six week-old mSOD (n = 3) and control (n = 3) mice were subjected to 10 Gy of ionizing radiation and transplanted with 5×10^∧^6 BM cells harvested from GFP-expressing mice. Blood was analysed at 3 weeks post-transplant to determine levels of peripheral blood cell chimerism.

### Nonirradiative Myeloablation

Control and presymptomatic mSOD mice aged 7–9 weeks were used for the following experiments. The chemotherapeutic drug BU for injection (Busulfex, Otsuka Pharmaceuticals, Japan) was diluted from the pharmaceutical stock solution to a concentration of 3 mg/ml using sterile PBS. For the short-term PBC chimerism and spinal cord BMDC accumulation experiments, control mice were given BU at doses of 60 (n = 4), 80 (n = 4), or 100 mg/kg (n = 3) BU. Twenty-four hours following the final dose of BU, mice received intravenous injections of 15×10^6^ GFP+ whole BM cells. Weekly blood samples were collected beginning at 1 week-post transplant. Mice were sacrificed and spinal cords harvested 4 weeks post-BM transplant for analysis of BMDC accumulation.

For the long-term analysis of PBC chimerism and BMDC accumulation in the spinal cord, mice were given BU at either 60 (n = 4 mSOD, 4 control) or 80 mg/kg (n = 4 mSOD, 4 control) IP in fractionated doses of 20 mg/kg per day. Twenty-four hours after the final BU treatment, mice received intravenous (IV) injections of 15×10^6^ GFP+ whole BM cells. mSOD and age-matched control mice were sacrificed when mSOD mice reached advanced disease stages, as defined by mice exhibiting a severe rolling gait, dragging hind limbs, or being unable to right themselves’ from a laterally recumbent position within 10 seconds. Weekly blood samples were collected beginning at 1 week post-transplant until 11 weeks post-transplant, a time point at which mSOD mice were reaching advantaged stages of disease and were sacrificed. Blood was immunolabeled with lymphoid (CD3 for T-cells, B220 for B-cells; conjugated to PeCy7 fluorophore) and myeloid (Gr1 for granulocytes, CD11b for monocytes; conjugated to APC fluorophore) lineage markers to analyze PBC chimerism. Analyses were performed using a BD Aria FACS machine (Becton-Dickenson, NJ, USA) to determine the proportion of donor-derived PBCs, as described previously [2].

An untreated-control group of mSOD mice (n = 3) and age-matched controls (n = 3) were sacrificed when mSOD mice reached advanced stages of disease. The number and types of T-cells within lumbar spinal cord sections were assessed and compared to mSOD and control mice that had been treated with 60 mg/kg BU or 80 mg/kg BU to determine if treatment with BU elicited/modified the number of T-cells seen in the CNS in control/mSOD mice.

### Tissue Processing

Mice were euthanized using CO2 and immediately transcardially perfused with 30 mL of 1×PBS followed by 30 mL of 4% paraformaldehyde (w/v; PFA). For BM chimeric mice a femur was collected following perfusion with PBS in order to assess levels of BM reconstitution by donor cells using flow cytometry. The spinal cord was dissected out, post-fixed in 4% PFA overnight at 4°C, and then immersed in 20% sucrose (w/v) in PBS at 4°C overnight for cryoprotection. After cryoprotection, tissue was embedded in TissueTek O.C.T. (Sakura Finetek, USA) and stored at −80°C until being cryosectioned at 30 µm as previously described [2].

### Immunohistochemistry

Free-floating spinal cord sections underwent immunohistochemical analysis as previously described [4]. To identify macrophages, antibody to the ionized Ca2+ -binding adapter (Iba1; Wako, VA, USA) was used; vascular endothelium was labelled using antibody to CD31 (PECAM1; BD Pharmingen, San Diengo, CA). Monocytes were identified using antibody to CD11b (Serotec, Raleigh, NC), granulocytes using antibody to Gr-1, activated phagocytes using antibody to CD68, and antibodies to CD3 and CD8 (BD Pharmingen, San Diego, CA) were used to identify and classify T-lymphocytes. Secondary antibodies used were either anti-rabbit Cy3-conjugated IgG (Iba1 visualization; Jackson Immunoresearch, West Grove, PA) or anti-rat Alexa568-conjugated IgG (CD3, CD8, CD31, Gr-1, CD68 visualization; Life Technologies, Burlington, ON). Immunolabeled sections were slide mounted and cover-slipped using Vectashield mounting medium (Vector Labs, Burlingame, CA*).*

### Analysis

Spinal cord sections were analyzed using a Leica epifluorescence microscope and a Nikon laser scanning confocol microscope. GFP+ cells from each mSOD and control mouse were quantified over 15 lumbar spinal cord sections separated by at least 150 µm and were classified according to morphology as previously described [4]. Briefly, GFP+ cells that were round in shape and <9 µm in diameter were classified as “round” while round cells with diameter >9 µm were classified as “amoeboid”. Cells were classified as “rod-shaped” if they were oblong, had rounded ends and a length of ∼20 µm. GFP+ cells were classified as elongated if their length exceeded 20 µm and did not have smooth/rounded ends. Stellate cells were identified by their small cell body and the presence of multiple ramified processes. The phenotypes of GFP+ BMDCs were analyzed using immunohistochemistry, and the numbers of Iba1+ and CD3+ BMDCs were quantified over 5 lumbar spinal cord sections from each experimental animal; all results are reported as mean ± standard deviation. For irradiative myeloablation, 5 lumbar spinal cord sections from each mSOD and control mouse were analysed and the number of GFP+ cells quantified by morphology.

The association of GFP+ cells with CD31 labelled blood vessels in spinal cord was assessed using a Nikon confocal microscope. Briefly, z-stack images of areas of interest in spinal cord sections were acquired to create a 3D rendering of the image. The volume rendering was then rotated about the x,y.z axes to visualize the assocaition of elongated GFP+ cells with blood vessels.

Quantitative assessment of GFP+ cells within the spinal cords of mSOD and control mice was statistically evaluated using SPSS software using an ANOVA; significance was taken at p<0.05.
